# Minor Trauma, Major Discovery: The Ultrasound Identification of a Sternal Fracture Missed by Computed Tomography in an Older Patient Presenting With Acute Chest Pain

**DOI:** 10.7759/cureus.61472

**Published:** 2024-06-01

**Authors:** Kazuki Miyaue, Hiroki Isono

**Affiliations:** 1 Department of General Medicine, HITO Medical Center, Ehime, JPN

**Keywords:** thoracic cage injury, elderly trauma, differential diagnosis, ultrasound (u/s), s: osteoporosis, acute chest pain, sternal fractures

## Abstract

This case report presents the diagnostic difficulties encountered by a 96-year-old woman with osteoporosis who experienced acute chest pain following minor trauma, eventually diagnosed with a sternal fracture. It highlights the nuanced presentation and diagnostic challenges associated with sternal fractures in older patients. Despite the prevalent use of chest radiography and computed tomography in acute trauma assessments, this case emphasizes their limitations, as both modalities initially failed to detect the sternal fracture. The successful identification of the fracture using ultrasound (US) underscores the utility of this modality in detecting subtle yet clinically significant injuries. This report advocates for a high index of suspicion for sternal fractures in older patients presenting with chest pain after minor trauma and suggests that US is a valuable, less invasive diagnostic tool. By illuminating the potential for minor trauma to cause major injury and the critical role of US in diagnosis, this case provides valuable insights into the management of sternal fractures in the geriatric population, urging clinicians to consider atypical presentations in diagnostic evaluations.

## Introduction

Sternal fractures, traditionally associated with significant trauma, primarily reflect the energy absorbed by the thoracic cage during events, such as motor vehicle collisions, where deceleration forces predominate [1]. The incidence of sternal fractures in these contexts ranges from 3% to 6.8%, underscoring their relevance in acute trauma care settings [1]. The biomechanical spectrum of sternal injuries distinguishes direct trauma from indirect trauma, with direct trauma predominantly arising from blunt anterior chest impacts and indirect trauma from mechanisms such as severe thoracic kyphosis or pathological weakening of the bone structure, notably in conditions such as osteoporosis or systemic diseases that affect bone density [1].

Chest radiography (CXR) is often the first choice for the diagnosis of sternal fractures; however, its sensitivity is low [2]. Computed tomography (CT) is the gold standard for diagnosing sternal fractures, with a better diagnostic performance than that of CXR [3].

Here, we present the case of an older woman with osteoporosis who presented with chest pain after a minor trauma and was eventually diagnosed with a sternal fracture by ultrasound (US).

## Case presentation

A 96-year-old woman presented to the emergency department with a distressing episode of chest pain exacerbated by a movement that started two days prior to her visit. The onset of her discomfort was traced to an innocuous backward fall observed a few days earlier. Her medical history included osteoporosis, lumbar fractures, Alzheimer’s disease, and chronic heart failure.

**Figure 1:**
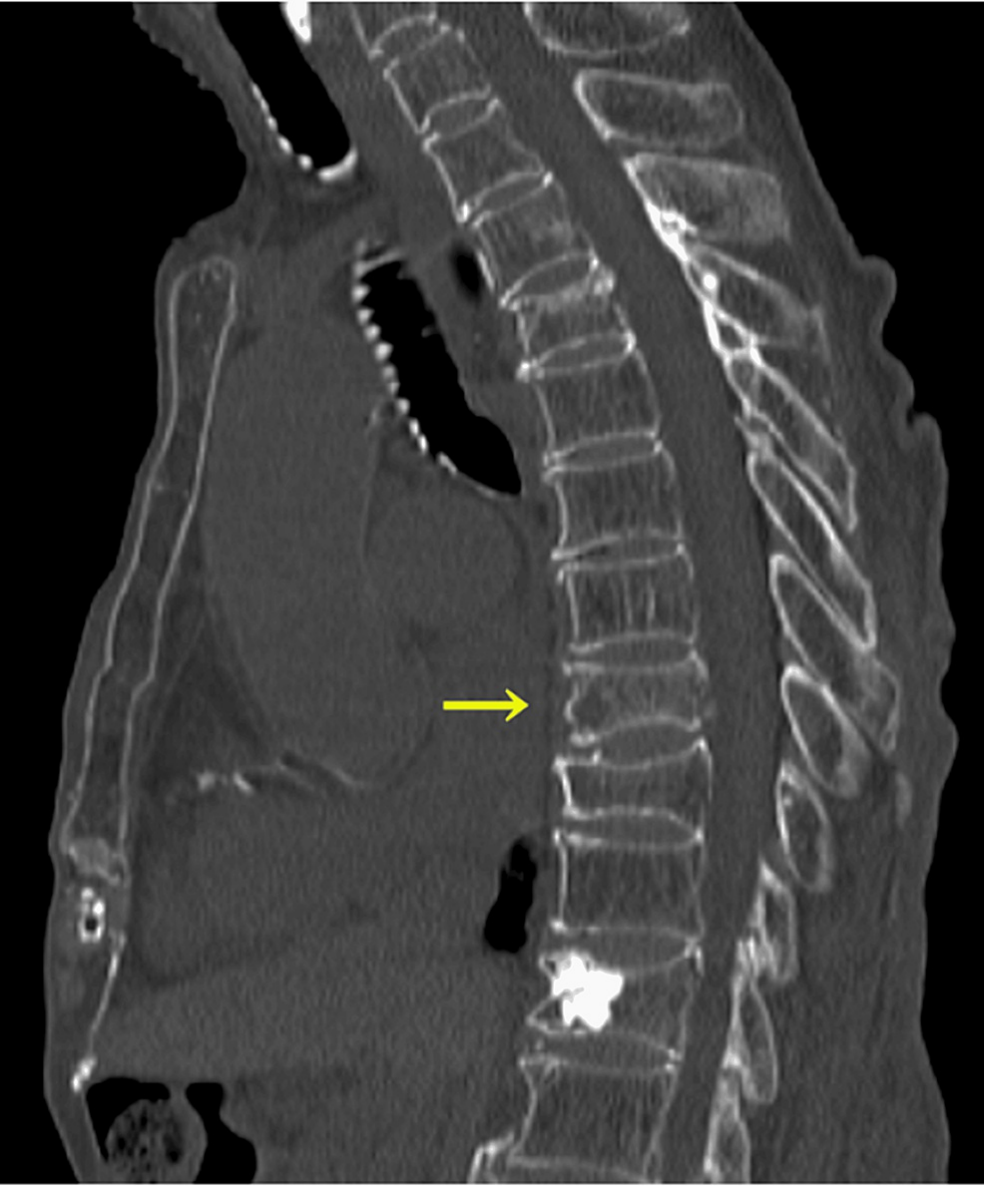
Computed tomography showing a thoracic spine fracture (yellow arrow).

**Figure 2:**
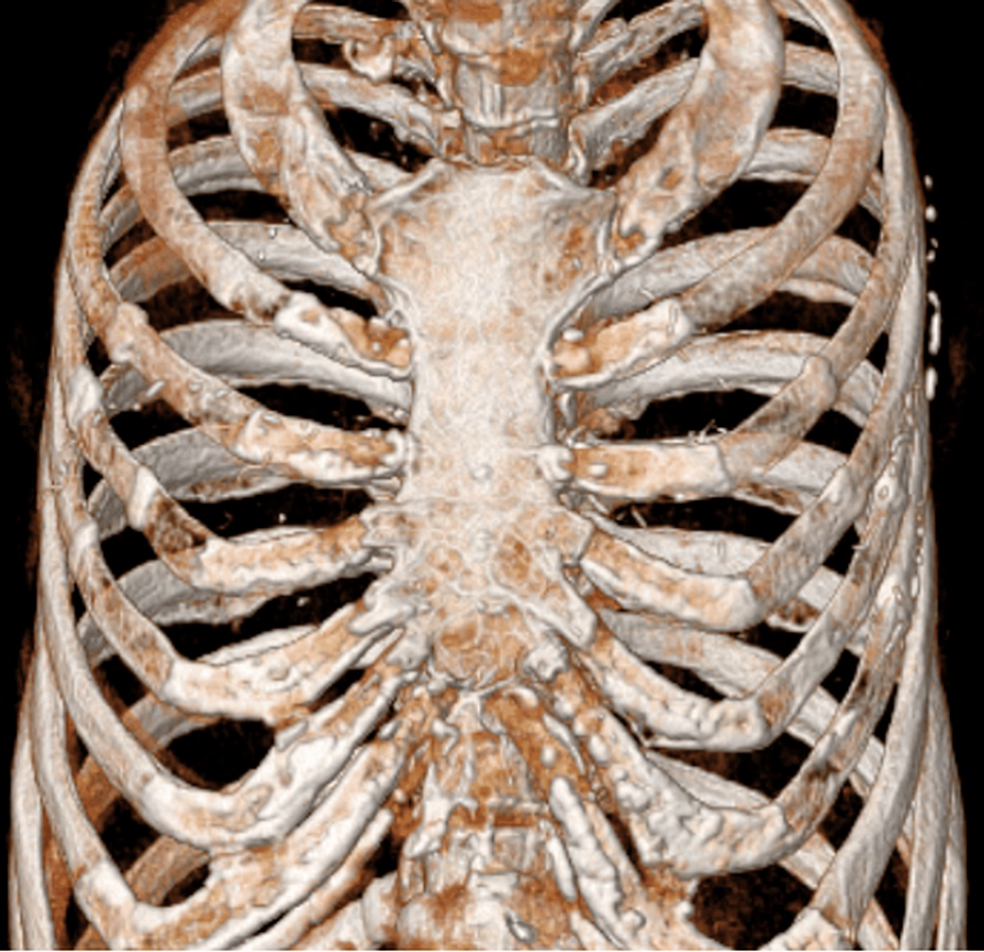
Computed tomography showing no apparent sternal fractures.

Her vital signs were within normal limits. The patient exhibited tenderness over the sternum. An anteroposterior CXR showed no abnormalities. The electrocardiogram results were also unremarkable. A CT scan of the chest revealed a thoracic spine fracture (Figure 1). However, the radiology report did not point out any other abnormalities that might cause her symptoms (Figures 1-2).

**Figure 3:**
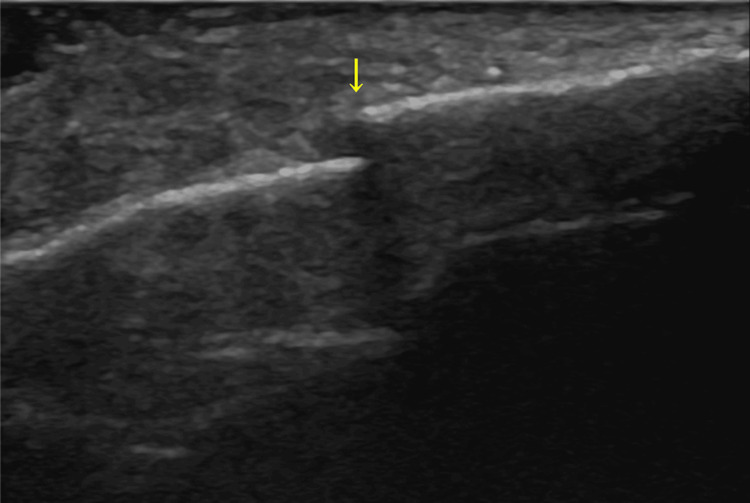
Ultrasound showing a sternal body fracture (yellow arrow).

The patient was then admitted for further evaluation. After admission, the patient had localized reproducible tenderness over the sternum, for which a sternal fracture was suspected. The chest US showed a sternal body fracture (Figure 3).

**Figure 4:**
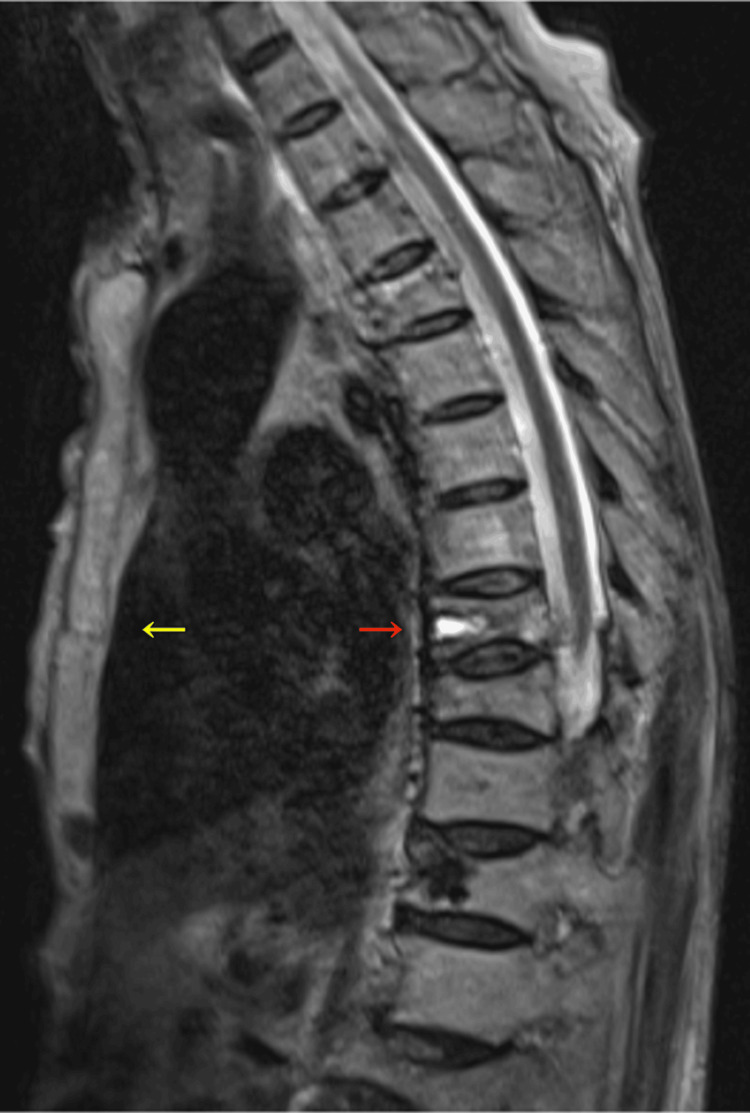
T2-weighted magnetic resonance imaging showing a sternal fracture (yellow arrow) and a thoracic spine fracture (red arrow) at the same segmental level.

Furthermore, the magnetic resonance imaging of the chest revealed sternal and thoracic spinal fractures at the same segmental level (Figure 4).

**Figure 5:**
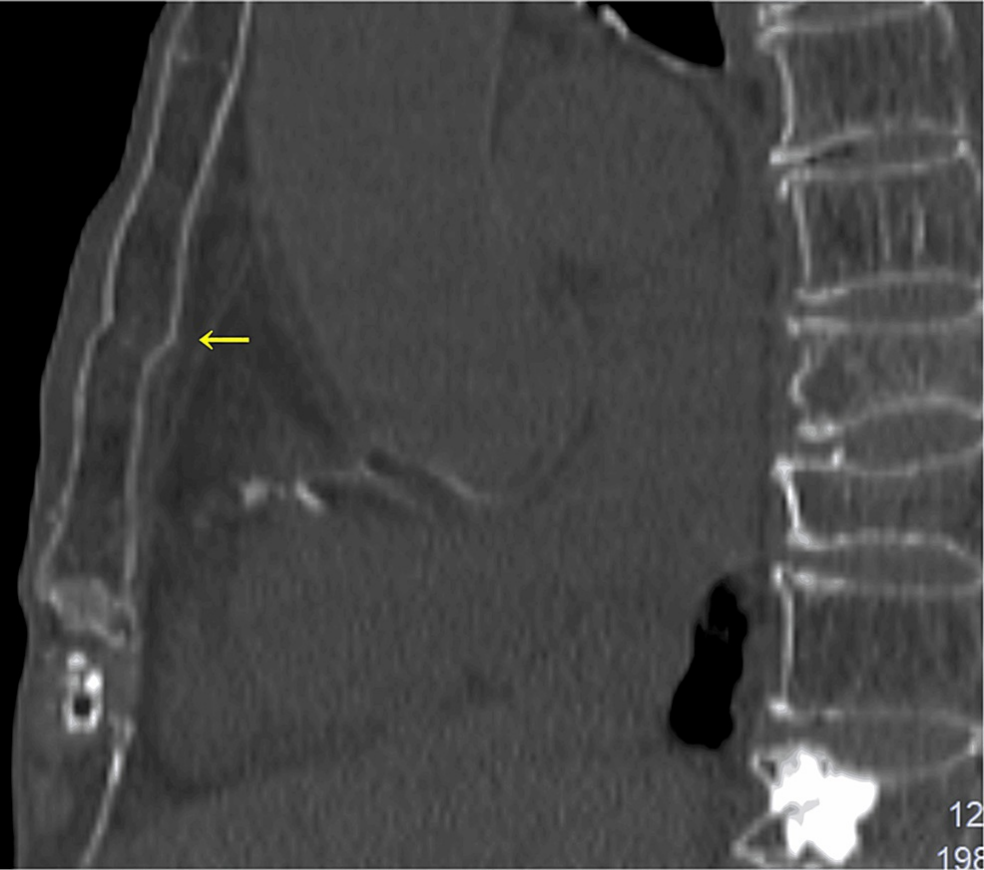
A re-review of computed tomography obtained in the emergency department indicating a sternal fracture (yellow arrow).

A re-review of the CT obtained at the emergency department revealed findings suggestive of a sternal fracture (Figure 5).

After consultation with orthopedic specialists, a conservative management strategy prioritizing pain alleviation and functional recovery without surgical intervention was suggested. The patient was discharged 24 days after rehabilitation.

## Discussion

Three pivotal findings were observed in the present case. First, this case highlights the possibility that CT may miss sternal fractures, a finding that emphasizes the diagnostic precision of US in this scenario. Second, it elucidates the phenomenon in which sternal fractures are precipitated by minor or even unperceived trauma, broadening the differential diagnostic spectrum for acute chest pain. Third, it indicates the importance of the investigation for concomitant injuries in case of sternal fractures, particularly in older patients even after minor trauma.

Our initial finding, as demonstrated in our case, strongly supports the occurrence of sternal fractures that cannot be detected on CT. One study showed that CT was 100% sensitive in detecting sternal fractures on sagittal images [4]. However, this study indicates that axial and coronal CT cannot occasionally detect sternal fractures [4]. Additionally, a case report showed that a sternal fracture was detected by US, which was missed by chest CT [5], as in our case, and another study illustrated that US is 100% sensitive for detecting sternal fracture [6]. These findings indicate that US is less harmful, swifter, and could be more precise than CT in diagnosing sternal fractures.

Our second finding is clearly illustrated by the present case, showing that minor trauma can cause sternal fractures, which can complicate diagnosis. Our case demonstrates that minor trauma can cause sternal fractures in an older patient. It is said that sternal fractures caused by minimal trauma, which are called sternal insufficiency fractures, are rarely reported compared with those caused by major trauma [7]. The major risk factors for insufficiency fractures include osteoporosis, thoracic kyphosis [7], and malignancies [8]. Diagnosing sternal insufficiency fractures can be challenging, as caregivers can miss an episode of trauma. Furthermore, it can be atraumatic in patients with risk factors [8], which explains why it mimics myocardial infarction or pulmonary embolism in patients with acute chest pain [9]. In the present case, the cause of chest pain was inconclusive in the emergency department, which required admission and further evaluation. A high index of suspicion for sternal fractures is important in patients presenting with chest pain without apparent causes.

Our third finding is the importance of searching for concomitant injuries in patients with sternal fractures, especially in older patients, even after minor trauma. It is said that combined sternal fractures (sternal fractures with fractures at other sites) are more common than isolated sternal fractures in geriatric patients [10]. A study indicates that the co-occurrence of sternal fractures and thoracic spine fractures sometimes results in an unstable thoracic cage requiring surgical stabilization [11]. In our case, sternal and thoracic spine fractures were found at the same segmental level, but the condition was stable, and conservative management was chosen.

## Conclusions

We present a case of a sternal fracture following minor trauma diagnosed using US in an older patient with osteoporosis. It reinforces the use of US as a fast, less harmful, and accurate diagnostic approach for suspected sternal fractures, particularly when CT yields inconclusive results. It also underscores the importance of suspecting sternal fractures in older patients with predisposing factors presenting with chest pain without known causes, regardless of whether the patient has a history of trauma. Additionally, this case highlights the importance of investigating for concomitant injuries in older patients, as combined fractures can complicate management.
